# Evaluation of an institutional series of low-grade oncocytic tumor (LOT) of the kidney and review of the mutational landscape of LOT

**DOI:** 10.1007/s00428-023-03673-9

**Published:** 2023-10-17

**Authors:** Costantino Ricci, Francesca Ambrosi, Tania Franceschini, Francesca Giunchi, Alessia Grillini, Eugenia Franchini, Marco Grillini, Riccardo Schiavina, Francesco Massari, Veronica Mollica, Valentina Tateo, Federico Mineo Bianchi, Lorenzo Bianchi, Matteo Droghetti, Thais Maloberti, Giovanni Tallini, Maurizio Colecchia, Andres Martin Acosta, João Lobo, Kiril Trpkov, Michelangelo Fiorentino, Dario de Biase

**Affiliations:** 1 Pathology Unit, Maggiore Hospital-AUSL Bologna, Bologna, Italy; 2 Department of Medical and Surgical Sciences (DIMEC), University of Bologna, Via Massarenti 9, 40138 Bologna, Italy; 3 Pathology Unit, IRCCS Azienda Ospedaliero-Universitaria Di Bologna, Bologna, Italy; 4 Division of Urology, IRCCS Azienda Ospedaliero-Universitaria Di Bologna, Bologna, Italy; 5 Medical Oncology, IRCCS Azienda Ospedaliero-Universitaria Di Bologna, Bologna, Italy; 6 Urology Department, Maggiore Hospital-AUSL Bologna, Bologna, Italy; 7 Solid Tumor Molecular Pathology Laboratory, IRCCS Azienda Ospedaliero-Universitaria Di Bologna, Bologna, Italy; 8 Department of Pathology, IRCCS San Raffaele Scientific Institute, Milan, Italy; 9 Department of Pathology, Indiana University School of Medicine, Indianapolis, USA; 10 Department of Pathology, Portuguese Oncology Institute of Porto (IPOP), Porto, Portugal; 11 Cancer Biology and Epigenetics Group, Portuguese Oncology Institute of Porto (IPO Porto)/Porto Comprehensive Cancer Center (P.CCC), Research Center of IPO Porto (GEBC CI-IPOP)/RISE@CI-IPOP (Health Research Network), Porto, Portugal; 12 Department of Pathology and Molecular Immunology, ICBAS-School of Medicine and Biomedical Sciences, University of Porto (ICBAS-UP), Porto, Portugal; 13 Department of Pathology and Laboratory Medicine, Cumming School of Medicine, University of Calgary and Alberta Precision Laboratories, Calgary, Canada; 14 Department of Pharmacy and Biotechnology, University of Bologna, Bologna, Italy

**Keywords:** Low-grade oncocytic tumor, LOT, Kidney tumors, Oncocytic tumors, MTOR, TSC1, TSC2

## Abstract

The 2022 WHO classification of urinary and male genital tumors introduced several novel kidney entities exhibiting eosinophilic/oncocytic features with specific mutational backgrounds. Thus, molecular techniques, such as next-generation sequencing (NGS), became more commonly used for their evaluation. We studied 12 low-grade oncocytic tumors (LOT) of the kidney (from 11 patients), identified in a cohort of 210 eosinophilic/oncocytic renal tumors, diagnosed in our institution between October 2019 and May 2023, which represented 5.7% (12/210) of all eosinophilic/oncocytic renal tumors during this period. We reviewed their clinicopathologic, histologic, and immunohistochemical features, as well as their mutational profiles. We also reviewed the literature on NGS-derived data of LOT, by selecting papers in which LOT diagnosis was rendered according to the criteria proposed initially. Median age was 65 years (mean: 63.5; range 43–79) and median tumor size was 2.0 cm (mean: 2.2; range: 0.9–3.1). All tumors were positive for PAX8, CK7, and GATA3, and negative or focally positive for CD117/KIT. We found the following gene mutations: *MTOR* ((6/11), 54.5%)), *TSC1* ((2/11), 18.2%)), and 1 had both *NOTCH1* and *NOTCH4* ((1/11), 9.1%)). Wild-type status was found in 2/11 (18.2%) patients and one tumor was not analyzable. A review of 8 previous studies that included 79 LOTs revealed frequent mutations in the genes that regulate the mammalian target of rapamycin (mTOR) pathway: *MTOR* (32/79 (40.5%)), *TSC1* (21/79 (26.6%)), and *TSC2* (9/79 (11.4%)). Other mutated genes included *PIK3CA*, *NF2*, and *PTEN*, not typically known to affect the mTOR pathway, but potentially acting as upstream and downstream effectors. Our study shows that LOT is increasingly diagnosed in routine practice when applying the appropriate diagnostic criteria. We also confirm that the mTOR pathway is strongly implicated in the pathogenesis of this tumor mainly through *MTOR*, *TCS1*, and *TSC2* mutations, but other genes could also be involved in the pathway activation, especially in LOTs without “canonical” mutations.

**Supplementary Information:**

The online version contains supplementary material available at 10.1007/s00428-023-03673-9.

## Introduction

The differential diagnosis of renal tumors with eosinophilic/oncocytic features is broad and involves several well-known and recently described entities, spanning from benign to malignant tumors, and typically arising in sporadic, but also in syndromic settings [1–13]. The 5^th^ edition (2022) of the WHO classification of urinary and male genital tumors introduced a separate category of eosinophilic/oncocytic tumors named “*oncocytic and chromophobe renal tumors*” that included well-defined entities, such as oncocytoma and chromophobe renal cell carcinoma (ChRCC), as well as a subset of “*other oncocytic tumours of the kidney*”. This group included two emerging entities, “low-grade oncocytic tumor” (LOT) and “eosinophilic vacuolated tumor” (EVT), as well as tumors considered “hybrid oncocytic/chromophobe tumors” (HOCT), typically arising in a hereditary setting (e.g. Birt-Hogg-Dubé (BHD) syndrome), and a heterogeneous group of sporadic eosinophilic/oncocytic tumors with borderline features designated “oncocytic renal neoplasm of low malignant potential-NOS” [1–7]. In addition, the new WHO classification recognized the specific mutational alterations in a broader category of renal tumors with eosinophilic/oncocytic cytoplasm that include microphthalmia-associated transcription factor family translocation RCC (MiTF RCC: TFE3-rearranged RCC and TFEB-altered RCC), succinate dehydrogenase–deficient RCC (SDH-RCC), fumarate hydratase–deficient RCC (FH-RCC), as well as novel recognized entities, such as eosinophilic solid and cystic RCC (ESC RCC) and ALK-rearranged RCC [1–7]. The recognition of specific molecular alterations in a growing number of renal entities resulted in a routine use of molecular techniques for their evaluation, such as next-generation sequencing (NGS), array-comparative genomic hybridization, and fluorescence in situ hybridization (FISH). Such molecular diagnostic tools complement the morphologic and immunohistochemical evaluation, especially in challenging and difficult-to-classify cases for which the clinicopathologic, histologic, and immunohistochemical are insufficient for a definitive diagnosis [1–7, 11–13]. After the seminal descriptions of LOT in 2019 [3, 4], a rapidly growing body of literature has supported and validated LOT as a distinct renal entity [1–7, 14–30]. LOT exhibits an oncocytoma-like morphology with round to oval nuclei and focal perinuclear halos, as well as edematous areas with scattered or irregularly distributed cells (“boats in a bay” appearance), and a characteristic immunohistochemical profile (diffuse positivity for CK7 and absent expression of CD117/KIT) [1–7, 14–30]. LOT has been shown to have frequent genetic alterations affecting the mammalian target of rapamycin (mTOR) pathway [1–7, 14–30], while lacking other major chromosomal alterations, including chromosomal losses and gains. However, these mTOR genetic alterations are non-specific and are shared by several other “pink” tumors, such as ESC RCC, EVT, and RCC FMS [1–6, 13–30]. For example, Xia et al. recently found that LOT, ESC RCC, EVT, and a group of similar tumors that do not completely fulfil the criteria for these entities all show *TSC/MTOR* mutations and have distinct RNA clustering expression profiles that separates them from each other, and from all other recognized renal tumors [25–27].

In this study, we aimed to interrogate an institutional case series of LOT with two in-house-developed multi-gene NGS panels. We also performed a literature review to clarify the genetic landscape of LOT, focusing exclusively on NGS data and including only tumors diagnosed according to the original description of LOT by Trpkov and Hes [3, 4].

## Materials and methods

### Case series

We identified a total of 12 LOTs diagnosed at DIAP-Dipartimento InterAziendale di Anatomia Patologica, Bologna, Italy (Pathology Unit, Maggiore Hospital, AUSL Bologna, Bologna, Italy; IRCCS Policlinico Sant’Orsola-Malpighi, University of Bologna Medical Center, Bologna, Italy) between October 1, 2019 and May 1, 2023. We intentionally chose this starting date, as the first descriptions of LOT appeared in the literature in 2019, to evaluate a time frame during which the diagnosis of LOT started to be routinely rendered at our institution [3, 4]. Two uropathologists (M.F. and C.R.) reviewed the cases using the original diagnostic criteria: oncocytic/eosinophilic cells with low-grade nucleoli, no/or focal perinuclear halos, no significant nuclear irregularities, ubiquitous loose stromal areas, and uniform immunohistochemical profile (CK7 + and CD117/KIT-) as originally described by Trpkov and Hes and adopted in the GUPS consensus paper and the 2022 WHO classification (5^th^ edition) [1–4]. During the selected study period, a total of 1670 renal tumors (1588 (95.1%) in-house cases and 82 (4.9%) consult cases) and 210 oncocytic/eosinophilic renal tumors (183 (87.1%) in-house cases and 27 (12.9%) consult cases) have been diagnosed at our institution. All cases were diagnosed as LOT, except one, which was identified upon additional review of all oncocytic/eosinophilic renal tumors that were not initially diagnosed as LOT. This tumor was diagnosed initially on core biopsy as “oncocytic renal neoplasm, not further specified”, with the accompanying comment “if this biopsy sample is representative of the entire lesion, the appearances would be consistent with a LOT (compatible morphology present but without the hypocellular areas, and immune profile CK7 + , CD117/KIT-)”, according to the GUPS recommendation [3, 5]. After a review of the histologic and immunohistochemical profile (see “Results” and Table 1: *tumor #1*), this tumor was reclassified as LOT and was included in the study [3, 4, 40]. A review of the 210 oncocytic/eosinophilic renal tumors showed the following distribution of different histotypes: oncocytoma (148, 70.5%), ChRCC (38, 18.1%) (ChRCC (classic) 31, 14.8%; ChRCC-eosinophilic 7, 3.3%), LOT (12, 5.7%), unclassified oncocytic tumors/RCCs (5, 2.4%), HOCT (2, 0.9%), EVT (1, 0.5%), ESC RCC (1, 0.5%), MiTF RCC (1, 0.5%), SDH-RCC (1, 0.5%), FH-RCC (1, 0.5%).

**Table 1:** Clinicopathologic features and NGS results of the analyzed cohort

Patient number	Case number	Sex	Age (years)	Tumor localization	Dimension (cm)	Follow-up (months)	NGS results (p.)	NGS results (c.)	VAF	ACMG significance	Variant type	Molecular consequence
1	1	F	79	R	2.3	NA	NA					
2	2	M	71	R	0.9	NED (35)	NOTCH1 (p.Glu515Lys)*°	*c.1543G* > *A*	48*	VUS/LB*	SNV	Missense
							NOTCH4 (p.Asp272Gly)*°	*c.815A* > *G*	55*	VUS/LB*	SNV	Missense
3	3	F	74	R	3.1	NED (28)	*MTOR (p.Ala2420Val)°*	*c.7259C* > *T*	28	P	SNV	Missense
4	4	M	62	L	2.5	NED (23)	*MTOR (p.Ser2215Tyr)°*	*c.6644C* > *A*	29	P	SNV	Missense
5	5	F	65	R	1.8	NED (20)	*MTOR (p.Leu2427Gln)°*	*c.7280 T* > *A*	36	P	SNV	Missense
6	6	F	58	R	2.3	NED (19)	*MTOR (p.Ser2215Phe)°*	*c.6644C* > *T*	17	P	SNV	Missense
7	7	M	50	R	1.9	NED (12)	WT°					
8	8	M	70	R	2.3	NED (22)	WT°					
9	9	M	66	L	1.4	NA	*MTOR (p.Thr1977Lys)°*	*c.5930C* > *A*	36	P	SNV	Missense
10	10	M	43	L	2.1	NED (5)	*TSC1 (p.Leu852AlafsTer53)^°*	*c.2549_2552dup*	65	LP	INDEL	Frameshift insertion
	11			L	1.7		*TSC1 (p.Leu852AlafsTer53)^°*	*c.2549_2552dup*	78	LP	INDEL	Frameshift insertion
11	12	M	61	L	2.0	NA	*MTOR (p.Leu2427Gln)°*	*c.7280 T* > *A*	25	LP	SNV	Missense

^*^In December 2022, these variants were classified as “variant of unknown significance” (VUS) in the Varsome database, but in June 2023, both were reclassified as “likely benign” (LB)

*°MTOR* c.7528 + 2 T > C variant was detected (splicing-site) in all specimens. This variant is reported as “likely pathogenic” (LP) in Varsome database. However, we identified this variant also in 10 non-neoplastic specimens (normal colonic mucosa) and thus, we did not consider this substitution in our cohort

*^*The same *TSC1* mutation was detected in the non-neoplastic tissue, and the subsequent clinical-genetic examination confirmed a diagnosis of TSC

*NGS*, next-generation sequencing; *VAF*, variant allele frequency; *ACMG*, American College of Medical Genetics and Genomics; *F*, female; *M*, male; *L*, left; *R*, right; *NA*, not available; *NED*, not evidence of disease; *WT*, wild-type status; *P*, pathogenic; *LP*, likely pathogenic; *LB*, likely benign; *VUS*, variant of unknown significance; *SNV*, single-nucleotide variant; *INDEL*, insertion-deletion; *LOT/LOTs*, low-grade oncocytic tumor/tumors; *TSC*, tuberous sclerosis complex (syndrome)

All clinicopathologic investigations were conducted according to the principles of the Declaration of Helsinki and all information regarding the human material used in this study has been managed using anonymous numerical codes. The study has been approved by the Review Board of the Area Vasta Emilia Centro-AVEC (IRB approval 3386/2018protocol). All patients included in the study provided informed consent, after a consultation with the investigators.

### *Clinicopathologic data and immunohistochemistry*

Clinical data (age, gender, tumor localization, and possible hereditary history) were collected from the digital records of the Urology Department (Maggiore Hospital-AUSL Bologna), Division of Urology (IRCCS Azienda Ospedaliero-Universitaria di Bologna), and Medical Oncology (IRCCS Azienda Ospedaliero-Universitaria di Bologna). Regardless of the initial immunohistochemistry work-up at the time of the original diagnosis, an additional evaluation was performed in all cases using the following marker panel: PAX8, CK7, CD117/KIT, GATA3, carbonic anhydrase-IX (CA-IX), CD10, alpha-methylacyl-CoA racemase (AMACR), CK20, cathepsin-K, fumarate hydratase (FH), succinate dehydrogenase B (SDHB), and transcription factor E3 (TFE3). The immunohistochemistry was performed using a BenchMark ULTRA automated immunostainer (Ventana Medical Systems-Roche Diagnostics, Switzerland). All stains were scored using the percentage of immunoreactive tumor cells, as follows: 0 = negative staining; 1 +  =  < 5% cells staining; 2 +  = 5–50% cells staining; 3 +  =  > 50% cells staining [22]. Expected nuclear and/or cytoplasmic reactivity of non-lesional cells (normal renal tubules, glomeruli, fibroblasts, endothelial cells, inflammatory cells, etc.) was used as internal controls. Clone antibodies, dilutions, and other technical data are summarized in Supplementary Material 1-Table S1.

### Molecular genetic analysis using a custom-designed NGS panel

DNA from formalin-fixed paraffin-embedded blocks was extracted using two to four 10-μm sections, under microscopic guidance from the representative tumor areas, identified by a pathologist on H&E slide. Extracted DNA was used for amplicon library preparation using two laboratory-developed multi-gene panels. The first panel (panel 1) allowed amplifying a total of 623 amplicons (69.7 kb, human reference sequence hg19/GRCh37) of the following genes: *AKT* (whole coding sequence (CDS)), *BRAF* (exons 11, 15), *EP300* (CDS), *HRAS* (exons 2–4), *KRAS* (exons 2–4), *MEN1* (CDS), *MTOR* (CDS), *NOTCH1* (CDS), *NOTCH2* (CDS), *NOTCH3* (CDS), *NOTCH4* (CDS), and *NRAS* (exons 2–4). The second panel (panel 2) allowed amplifying a total of 280 amplicons (29.24 kb, human reference sequence hg19/GRCh37) in the whole CDS of the following genes: *FH*, *FOXL2*, *HMGA1*, *MED12*, *TSC1*, and *TSC2*. Briefly, about 30 ng of input DNA was used for NGS library preparation for each panel using the AmpliSeq Plus Library Kit 2.0 (Thermo Fisher Scientific, Waltham, MA, USA). Templates were then sequenced using an Ion 530 chip and the results were analyzed with the IonReporter tools (version 5.18, Thermo Fisher Scientific) and GenomeBrowser Tool (https://www.goldenhelix.com/). According to the previously reported validation, only mutations present in at least 5% of the total number of analyzed reads and observed in both strands were considered as mutational calls [31]. The Varsome tool (https://varsome.com/, updated to April 2023) was used to evaluate the American College of Medical Genetics and Genomics classification of each mutation [32].

### Literature review of mutational landscape of LOT—selection criteria

We reviewed the literature on the mutational profile of LOT to select only studies encompassing NGS data and published by April 2023, in line with the study time frame and selection criteria. We only selected studies in which the diagnosis of LOT had been rendered using the histomorphologic and immunohistochemical criteria proposed by Trpkov and Hes [3, 4]. In our review, we excluded tumors with absent and/or incomplete clinicopathologic and immunohistochemical data (CK7, CD117/KIT), in which a definitive diagnosis of LOT could not be established, using the accepted criteria [19, 33–40]. Indeed, many LOTs have likely been reported in the literature and in The Cancer Genome Atlas (TCGA) as other entities (for example, as oncocytomas and ChRCC-eosinophilic) and we did not include such tumors because a definitive diagnosis of LOT could not be established [19, 33–40].

## Results

### Clinicopathologic data

A total of 12 LOTs obtained from 11 patients were evaluated; patient #10 showed two distinct tumors (*tumors #10* and *#11*) in the left kidney. Overall, during the study period, these 12 LOTs represented 0.7% (12/1670) of the institutional renal tumor case volume and 5.7% (12/210) of all oncocytic/eosinophilic renal tumors. In 10 (83.3%) patients, partial nephrectomy was done, 1 (8.3%) had radical nephrectomy, and 1 (8.3%) had a core biopsy. Patient median age was 65 years (mean: 63.5; range 43–79), and they were more commonly male (7/11, 63.6%). None of the patients had a clinical history of BHD, TSC, oncocytosis, or other hereditary conditions. The median tumor size on gross examination was 2.0 cm (mean: 2.2; range: 0.9–3.1) and all appeared organ-confined. Follow-up was available in 8/11 (72.7%) patients (mean 20.5 months; median 21 months; range 5–35 months) and all patients were alive and without evidence of disease. Clinicopathologic data are summarized in Table 1.

### Morphology and immunohistochemical data

**Fig. 1:**
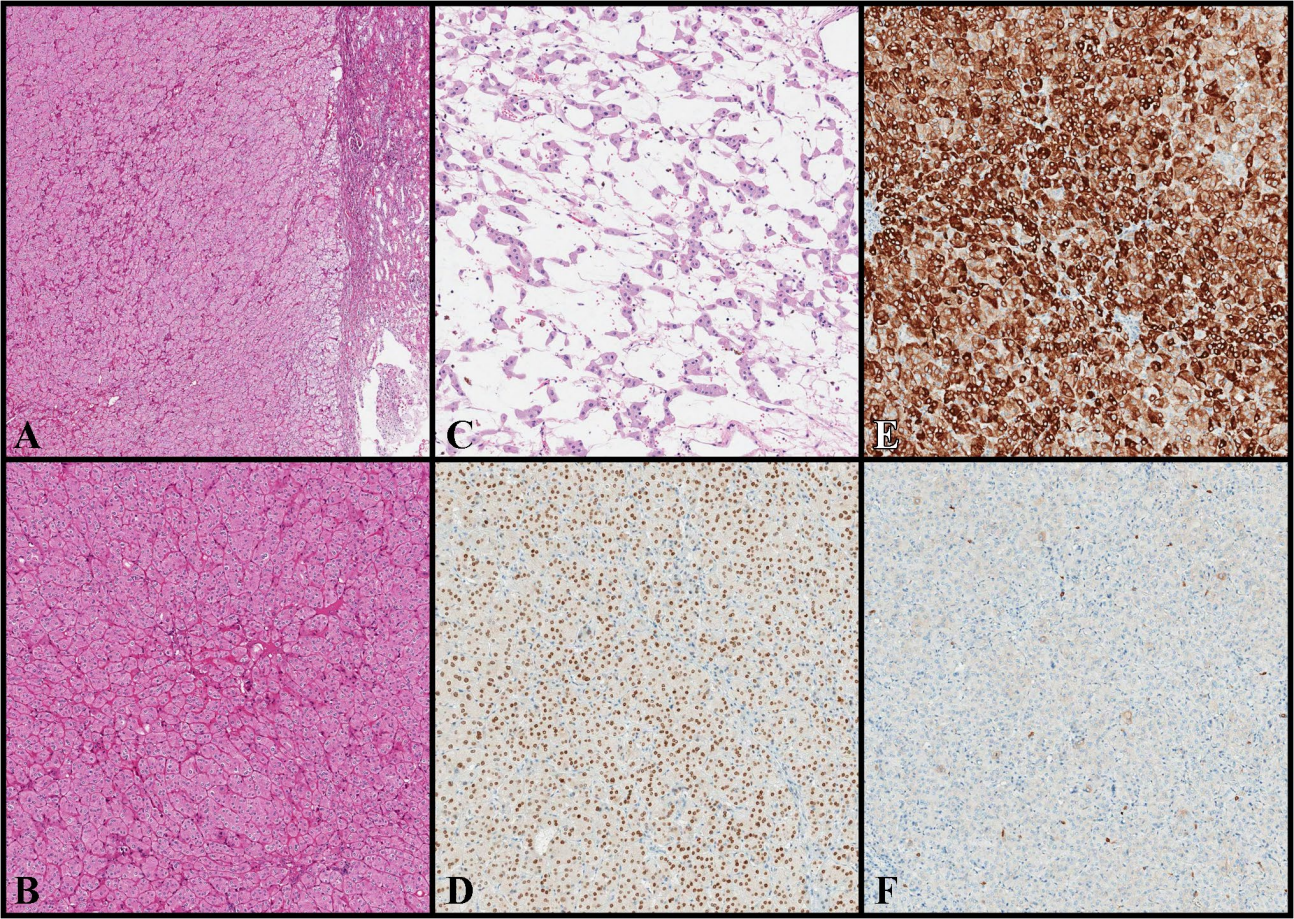
Histologic and Immunohistochemical features of LOT. At low-power magnification (**A**, H&E, original magnification 50 ×), LOT shows an oncocytoma-like appearance with a prominent solid architecture, absence of peripheral capsule, and rare entrapped renal tubules at the periphery. At high-power magnification (**B**, H&E, original magnification 100 ×), LOT displays oncocytic/eosinophilic cells with low-grade nucleoli, absence of irregular/raisonoid nuclear appearance, and no significant perinuclear halos. In the central area, LOT exhibits the so-called boats in a bay arrangement with hypocellular areas occupied by loosely arranged tumor cells (**C**, H&E, original magnification 100 ×). At immunohistochemistry, LOT results positive for GATA3 (**D**, original magnification 100 ×) and CK7 (**E**, original magnification 100 ×), but negative for CD117/KIT (**F**, original magnification 100 ×). *The* *chromogen (labeling) for GATA3, CK7, and CD117/c-kit is DAB*

All tumors showed an oncocytoma-like appearance with a prominent solid architecture and focal nested-tubular arrangement. Noteworthy, all tumors showed loose stromal areas with hypocellular and irregular cell distribution, often resembling tissue culture (with cell cords, reticular structures, and individual scattered cells); such areas often contained fresh hemorrhage. The tumors lacked a peripheral capsule but displayed rare entrapped renal tubules at the periphery. Scattered lymphocytic clusters were observed in the solid areas, often forming round to oval, more compact aggregates. The cells had low-grade nuclei (equivalent to WHO/ISUP grade 2/4) with focal, delicate perinuclear halos. and did not show irregular or raisinoid nuclear appearances. Although in *tumor #1* (core biopsy), the architecture was not evaluable, the cells exhibited similar features. Adverse or worrisome histological features, including lympho-vascular invasion, necrosis, marked nuclear pleomorphism, multinucleated cells, and mitoses, were absent in all tumors. All tumors were diffusely positive for PAX8, CK7, and GATA3 (3 + : 12/12 (100%)), and negative or focally positive for CD117/KIT (0: 8/12 (66.7%), 1 + : 4/12 (33.3%)). Cathepsin-K was negative (0: 8/12 (66.7%)), or focally positive (1 + : 4/12 (33.3%)). CA-IX, CK20, and TFE3 were all uniformly negative (0: 12/12 (100%)), whereas CD10 (0: 10/12 (83.3%), 1 + : 2/12 (16.7%)) and AMACR (0: 9/12 (75%), 1 + : 3/12 (25%)) were focally expressed in some tumors. FH and SHDB were retained in all tumors (2 + : 12/12 (100%)). Immunohistochemical results are summarized in Supplementary Material 2-Table S2. An illustrative example of LOT (H&E and relevant immunohistochemistry) is shown in Fig. 1.

### Molecular results

**Fig. 2:**
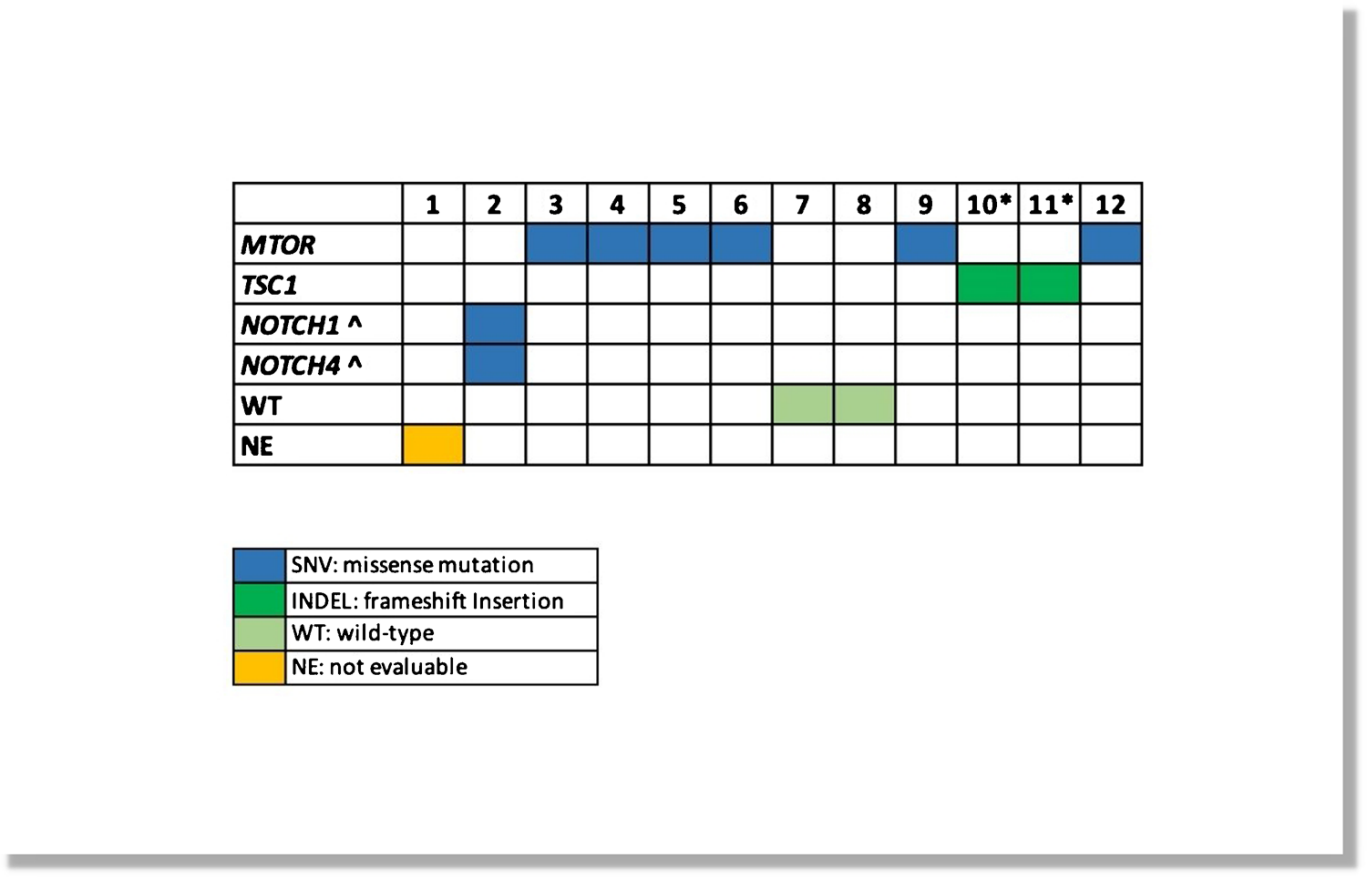
Genomic profile of the LOTs in the cohort. These two specimens (indicated by asterisk symbols) were obtained from one patient and the analysis on the non-neoplastic tissue revealed the same *TSC1* substitution. At December 2022, these variants (indicated by upward-pointing arrowheads) were classified as VUS in the Varsome database, but in June 2023, both were reclassified as likely benign

Of the 12 samples analyzed by NGS (Fig. 2), one was not evaluable due to low-quality DNA. Of the remaining 11 specimens, 6/11 (54.5%) harbored *MTOR* mutations (5 pathogenic and 1 likely pathogenic variants) and 2/11 (18.2%) *TSC1* mutations (with identical likely pathogenic variant); of note, all mutations were found in different specimens with no overlap (Fig. 2). Notably, two LOTs (*tumors #10* and *#11*) with identical *TSC1* mutations were obtained from one patient; analysis of the non-neoplastic tissue of this patient had the same *TSC1* substitution, which strongly suggested a diagnosis of TSC (Table 1 and Fig. 2). This patient underwent clinical-genetic examination which confirmed the diagnosis of TSC; the patient had epileptic episodes due to multiple cortical tubers and had skin hamartomas. Of note, in 1/11 (9.1%) tumors, there were two co-occurring mutations: *NOTCH1* mutation (p.Glu515Lys) and *NOTCH4* mutation (p.Asp272Gly). While in December 2022 these variants were classified as variants of unknown significance (VUS) in the Varsome database, in June 2023, both were reclassified as likely benign.

### Literature review of the mutational landscape of LOT

Using the outlined criteria, 8 previous studies were identified for review, resulting in a total of 79 LOTs tested by NGS panels, including tumors from the current study (summarized in Tables 2 and 3) [20–25, 28, 29]. The most frequently detected mutations were those typically known to affect the mTOR pathway: *MTOR* (32/79 (40.5%)), *TSC1* (21/79 (26.6%)), and *TSC2* (9/79 (11.4%)) (Tables 2 and 3). Two tumors (2.5%) showed two different *TSC1* mutations, and 5 tumors (6.3%) had co-mutations in two of these three genes (2 tumors with *MTOR* and *TSC2* mutations, and 3 tumors with *TSC1* and *TSC2* mutations). However, other genes, “not typically known” to affect the mTOR pathway, such as *STK11*, *PTEN*, *FOXP1*, *FGFR3*, *NF2*, *MET*, *PIK3CA*, *RHEB*, *CDKN2A*, *EZH2*, *SETD2*, and *PIK3CA*, were also mutated in these tumors (Table 2). These genes may be potentially involved in the abnormal activation acting as upstream and/or downstream effectors of the mTOR pathway. Of note, 12/24 (50%) of these alternative mutations were not concomitant with *MTOR*, *TSC1*, and *TSC2* mutations. Notably, *PIK3CA* was not concomitant and was found as pathogenic in 5/6 (83.3%) tumors, which may suggest that *PIK3CA* mutation may be “sufficient” on its own to activate the mTOR pathway, without the concurrent *MTOR*, *TSC1*, and *TSC2* mutations. Overall, 14/24 (58.3%) of these alternative mutations were found as pathogenic and/or likely pathogenic (see Tables 2 and 3). There were 9/79 (11.4%) tumors with a wild-type (WT) status and without detectable mutations by the NGS panels used. However, due to variability and scope of the NGS panels used in different studies, we cannot rule out that other relevant undetected genes may have been implicated in these tumors.

**Table 2:** Mutational landscape of LOTs (our case series and review of the literature)

Author and study	Number of LOTs with evaluable NGS results	Number of LOTs with specific mutations (absolute number, % in relation to the number of LOTs)	Cases with co-mutations
*Tjota *et al*.*^*22*^	6	*TSC1* (4, 66.7%), *TSC2* (1, 16.7%), *MTOR* (1, 16.7%%)	Two different *TSC1* mutations (1)
*Mohanty *et al*.*^*24*^	14	*TSC1* (4, 28.6%), *TSC2* (1, 7.1%), *MTOR* (1, 7.1%), *STK11* (2, 14.3%), *PTEN* (1, 7.1%), *FOXP1* (1, 7.1%), *FGFR3* (1, 7.1%), *NF2* (1, 7%), *MET* (1, 7.1%), *PIK3CA* (1, 7.1%), wt (3, 21.4%)	*STK11* and *TSC1* (1); *PTEN* and *TSC1* (1); *MET* and *TSC1* (1)
*Kapur *et al*. and Durinck *et al*.*^*20,21*^	6	*MTOR* (4, 66.7%), *TSC1* (1, 16.7%), *RHEB* (1, 16.7%)	
*Xia *et al*.*^*25*^	8	*MTOR* (7, 87.5%), *TSC1* (1, 12.5%)	Two different *TSC1* mutations (1)
*Zhang *et al*.*^*23*^	7	*MTOR* (1, 14.3%), *TSC1* (1, 14.3%), *TSC2* (5, 71.4%), *CDKN2A* (1, 14.3%), *EZH2* (2, 28.6%), *SETD2* (2, 28.6%), *PIK3CA* (1, 14.3%), wt (2, 28.6%)	*MTOR*, *TSC2*, *CDKN2A*, and *EZH2* (1); *TSC1*, *TSC2*, and *SETD2* (2); *TSC1*, *TSC2*, *EZH2*, and *PIK3CA* (1); *TSC1* and *TSC2* (1)
*Morini *et al*.*^*28*^	10	*MTOR* (7, 70%), *TSC1* (1, 10%), wt (2, 20%)	
*Williamson *et al*.*^*29*^	17	*TSC1* (7, 41.2%), *TSC2* (2, 11.8%), *MTOR* (5, 29.4%), *PIK3CA* (4, 23.5%), *NF2* (2, 11.8%), *PTEN* (1, 5.8%)	*TSC2* and *NF2* (1); *TSC2* and *MTOR* (1); *MTOR* and *NF2* (1); *TSC1* and *PTEN* (1)
Our case series	11	*MTOR* (6, 54.5%), *TSC1* (2, 18.2%), *NOTCH1* (1, 9.1%), *NOTCH4* (1, 9.1%), wt (2, 18.2%)	*NOTCH1* and *NOTCH4* (1)

Eight previous studies [20–25, 28, 29] and a total of 79 LOTs (by adding our case series) were included in this review (see “Case series” in “Materials and methods” and “Results”)

*LOT*, low-grade oncocytic tumor; *WT*, wild-type status

**Table 3:** Mutational landscape of LOTs (our case series and review of the literature)

Gene	Number of LOTs with specific mutations, total number (%)	ACMG significance
*TSC1*	21 (26.6%)	P: 16, VUS: 2, NS: 5; 2 patients had 2 different *TSC1* mutation (first patient: 1 VUS and 1 P; second patient: 2 NS)
*TSC2*	9 (11.4%)	P: 3, LP: 1, VUS: 2, B: 1, B/VUS: 1, NS: 1
*MTOR*	32 (40.5%)	P: 16, P/LP: 1, LP: 3, VUS: 6, NS: 6
*STK11*	2 (2.5%)	P: 2
*PTEN*	2 (2.5%)	P: 2
*FOXP1*	1 (1.3%)	P: 1
*FGFR3*	1 (1.3%)	P: 1
*NF2*	3 (3.8%)	P: 2, LB/VUS: 1
*MET*	1 (1.3%)	P: 1
*PIK3CA*	6 (7.6%)	P: 4, P/LP: 1, NS: 1
*RHEB*	1 (1.3%)	NS: 1
*CDKN2A*	1 (1.3%)	NS: 1
*EZH2*	2 (2.5%)	NS: 2
*SETD2*	2 (2.5%)	NS: 2
*NOTCH1*	1 (1.3%)	VUS/LB: 1
*NOTCH4*	1 (1.3%)	VUS/LB: 1
WT	9 (11.4%)	

Eight previous studies [20–25, 28, 29] with a total of 79 LOTs (including current study) were included in the review

*LOT*, low-grade oncocytic tumor; *WT*, wild-type status; *ACMG*, American College of Medical Genetics and Genomics; *P*, pathogenic; *LP*, likely pathogenic; *NS*, not specified; *LB*, likely benign; *B*, benign; *VUS*, variant of unknown significance

## Discussion

**Fig. 3:**
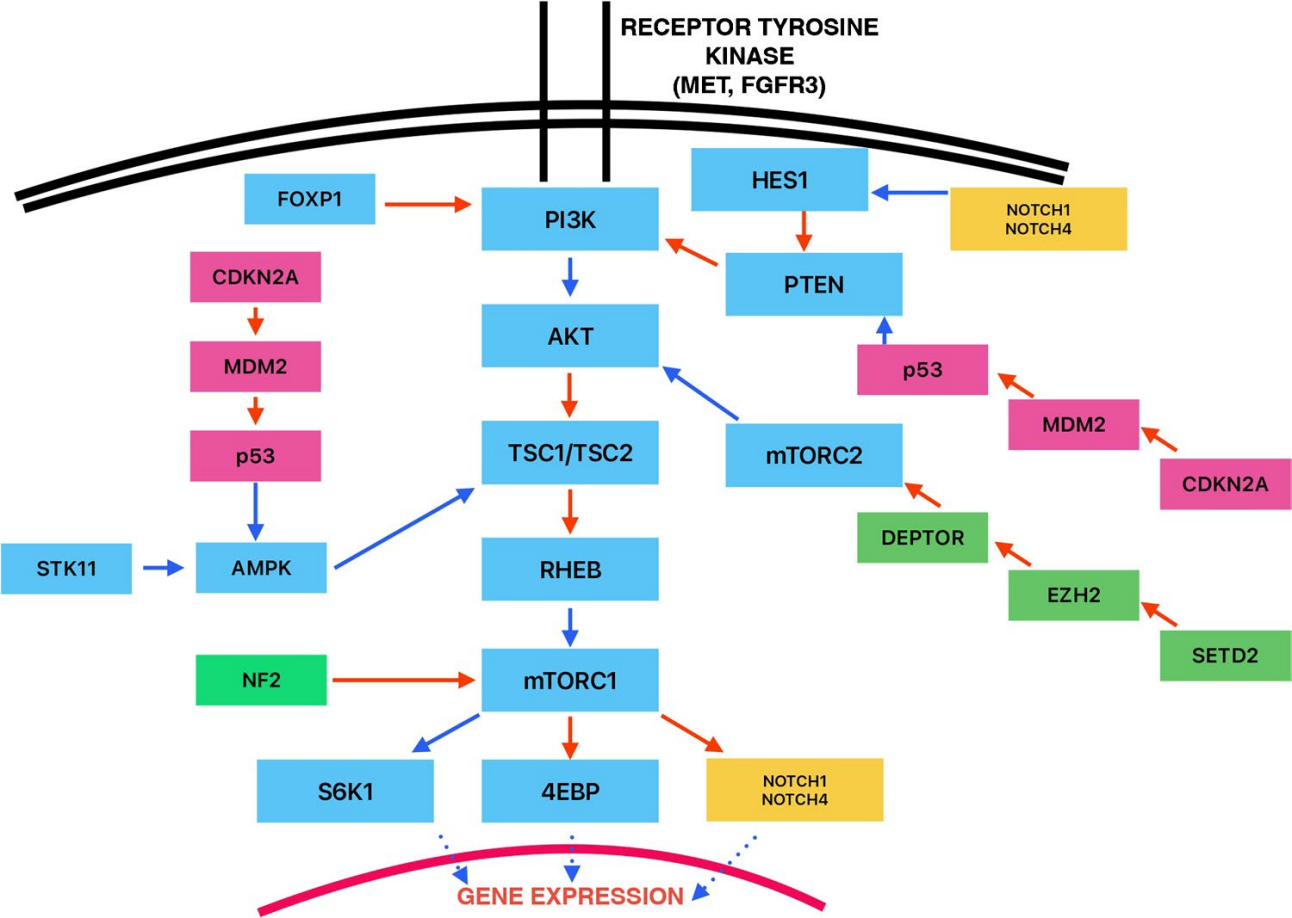
MTOR pathway with potential regulation mechanisms and genes mutated in LOT (based on current series and previous studies included in this review) [20–25, 28, 29]. Blue arrows indicate positive/activating signals, red arrows indicate negative/inhibitory signals, and dotted blue arrows indicate effects on gene expression (positive or negative depending on the targeted gene)

One of the more challenging areas in uropathology is the differential diagnosis of renal tumors with eosinophilic/oncocytic features [1–7]. An increasing number of studies included molecular evaluations that facilitated the recognition of several new entities, which were separated from the spectrum of tumors previously incorrectly labelled as oncocytoma, eosinophilic ChRCC, or considered “unclassified oncocytic tumors/RCCs” [1–7]. In recent years, mTOR pathway prompted increasing interest and focus of study as an underlying theme for several novel and emerging entities [1–7, 14–30, 33, 35–37]. mTOR was known to be involved in the pathogenesis of angiomyolipoma (both in syndromic and non-syndromic scenarios), but mutations in genes that regulate this pathway (*TSC1*, *TSC2*, and *MTOR*) were also rarely found in some common renal tumors, such as ChRCC, clear cell RCC, and papillary RCC [1–9, 37–39]. Nevertheless, the mTOR pathway was found more recently to be primarily involved in the pathogenesis of specific “pink tumors”, such as ESC RCC, EVT, LOT, and RCC FMS [1–7, 14–30, 33]. The shared molecular alterations affecting the mTOR pathway led some authors to wonder if these tumors should be grouped as “*TSC*/*MTOR*-associated renal tumors”, although they demonstrated different clinicopathologic, histologic, and immunohistochemical features [25–27]. However, the presence of distinct morphologic and immunohistochemical features, as well as the differences in gene involvement in different entities (for example, ESC RCC showed almost exclusively bi-allelic somatic *TSC2* mutations) led some authors to conclude that although these neoplasms share mTOR pathway molecular mechanisms, they should be considered as distinct entities [25–27]. In the present study, we evaluated a case series of LOT of the kidney with a laboratory-developed multi-gene NGS panel. We intentionally selected a time frame following the original description of LOT to obtain more reliable data on the prevalence of this tumor in routine diagnostic practice [3, 4]. We identified 12 LOTs, which represented 0.7% (12/1670) of the total renal tumor volume and 5.7% (12/210) of all oncocytic/eosinophilic renal tumors. These frequencies are higher than those reported in previous studies and suggest that the application and familiarity with well-defined histologic and immunohistochemical criteria increases the number of LOT cases diagnosed in routine practice [17, 25, 27]. We also confirm a consistent and uniform GATA3 reactivity in all LOTs included in the current study, which supports its usefulness as an additional marker in the diagnosis of LOT [29, 40]. In our experience, GATA3 can be also variably and focally expressed in ChRCC, but it is essentially negative in all other eosinophilic/oncocytic renal tumors. Based on the 8 reviewed studies and an aggregate cohort of 79 LOTs, the majority (57/79 (72.1%)) exhibited at least one mutation in the three genes (*MTOR*, *TSC1*, and *TSC2*), well-known to be involved in the activation of the mTOR pathway; *MTOR* (32/79 (40.5%)) was preferentially involved compared to *TSC1* and *TSC2*. An additional interesting finding is the relatively high mutation frequency (15/79 (19%)) found in other genes (*STK11*, *PTEN*, *FOXP1*, *FGFR3*, *NF2*, *MET*, *PIK3CA*, *RHEB*, *CDKN2A*, *EZH2*, *SETD2*, *NOTCH1*, and *NOTCH4*). Although these genes are known to be involved in distinct and well-defined intracellular mechanisms, they could also display promiscuous and heterogenous effects, potentially causing the up- and down-stream activation of the mTOR pathway (Fig. 3) [24, 29, 41–43]. Although it is difficult to establish specifically which of these mutations affect the mTOR pathway or are “simply passenger mutations”, 14/24 (58.3%) were found as pathogenic and/or likely pathogenic (Table 3). Moreover, 12/24 (50%) were not concomitant with the mutations in *MTOR*, *TSC1*, and *TSC2* (Table 2); among these genes, *PIK3CA* was pathogenic/likely pathogenic and not concomitant in 5/6 (83.3%) tumors. Kapur et al. analyzed the relationship between the detected mutations, the mTORC1 structure, and the intracellular levels of p-S6 and p-4EBP1 (specific markers of the mTORC1 activation) to evaluate the impact of the individual mutations on mTORC1 activity [21]. They found that levels of mTORC1 activation varied depending on the type of mutation (for example, mTORC1 activation was lower in tumors with *MTOR* mutations) [21]. These data, along with our findings, suggest that some of these “non-canonical” mTOR-activating mutations may be “sufficient” on their own to activate the mTOR pathway. A subset of mutations could also cooperate with “canonical” mTOR-activating mutations in determining and increasing the final degree of mTORC1 function (e.g. *tumor #2* in study by Mohanty et al. with *STL11* and *TSC1* mutations [24]). These results suggest that a subset of LOTs and especially tumors without detectable *MTOR*, *TSC1*, and *TSC2* mutations may have other mutated genes that may potentially activate the mTOR pathway.

To conclude, our results show that LOT is a straightforward diagnosis in routine practice, typically requiring morphologic and limited immunohistochemistry evaluation, as outlined in the initial studies and in the current WHO classification [1–4]. This institutional LOT cohort assembled after the initial publications indicates that LOT appears to be more frequent than previously reported. Our review of the molecular landscape of LOT shows that the mTOR pathway is strongly implicated in the pathogenesis of LOT. In tumors with no mutations in *TCS1*, *TSC2*, and *MTOR* genes, mutations in other genes may potentially affect the mTOR pathway and additional studies utilizing broader and standardized molecular gene panels may further clarify this issue.

### Supplementary Information

Below is the link to the electronic supplementary material.

Supplementary file1 (DOCX 15 KB)

Supplementary file2 (DOCX 20 KB)

## Data Availability

The datasets generated during and/or analyzed during the current study are available from the corresponding author on reasonable request.
