# Supplementary material for: Evaluation of an institutional series of low-grade oncocytic tumor (LOT) of the kidney and review of the mutational landscape of LOT
Source: Virchows Arch. 2023 Oct 17;483(5):687–98. doi: 10.1007/s00428-023-03673-9 (PMC10673759; doi:10.1007/s00428-023-03673-9)
Supplement: Supplementary file 1 — Supplementary file1 (DOCX 15 KB) [file 428_2023_3673_MOESM1_ESM.docx]

**Supplementary Material 1 (Table S1) - Immunohistochemical clones, chromogens, and sources of the adopted antibodies**

CA-IX: carbonic anhydrase-IX; AMACR: alpha-methylacyl-CoA racemase; FH: fumarate hydratase; SDHB: succinate dehydrogenase B; TFE3: transcription factor E3; DAB: 3,3’-Diaminobenzidine (brown color);

| Antibody | Specie | Clone | Chromogen (labelling) | Source |
| --- | --- | --- | --- | --- |
| PAX8 | Rabbit (monoclonal) | EP331 | DAB | Cell Marque, USA |
| CK7 | Rabbit (monoclonal) | SP52 | DAB | Ventana, USA |
| CD117/KIT | Rabbit (monoclonal) | YR145 | DAB | Cell Marque, USA |
| CK20 | Rabbit (monoclonal) | SP33 | DAB | Ventana, USA |
| AMACR | Rabbit (monoclonal) | SP116 | DAB | Ventana, USA |
| CA-IX | Rabbit (monoclonal) | EP161 | DAB | Cell Marque, USA |
| CD10 | Rabbit (monoclonal) | SP67 | DAB | Ventana, USA |
| Cathepsin-K | Mouse (monoclonal) | 3F9 | DAB | Abcam, UK |
| FH | Mouse (monoclonal) | J-13 | DAB | Santa Cruz Biotechnology, USA |
| SDHB | Rabbit (polyclonal) |  | DAB | Sigma-Aldrich, USA |
| TFE3 | Rabbit (monoclonal) | MRQ-37 | DAB | Ventana, USA |
| GATA3 | Mouse (monoclonal) | L-50-823 | DAB | Ventana, USA |
