# Supplementary material for: Evaluation of an institutional series of low-grade oncocytic tumor (LOT) of the kidney and review of the mutational landscape of LOT
Source: Virchows Arch. 2023 Oct 17;483(5):687–98. doi: 10.1007/s00428-023-03673-9 (PMC10673759; doi:10.1007/s00428-023-03673-9)
Supplement: Supplementary file 2 — Supplementary file2 (DOCX 20 KB) [file 428_2023_3673_MOESM2_ESM.docx]

**Supplementary Material 2 (Table S2) - Immunohistochemical features of the case series**

All stains were scored regarding the percentage of immunoreactive tumor cells, as follows: 0 = negative staining; 1+ = <5% cell staining; 2+ = 5-50% cell staining; 3+ = >50% cell staining [22].

CA-IX: carbonic anhydrase-IX; AMACR: alpha-methylacyl-CoA racemase; FH: fumarate hydratase; SDHB: succinate dehydrogenase B; TFE3: transcription factor E3;

| Patient Number | Case Number | PAX8 | CK7 | CD117/  KIT | GATA3 | CA-IX | CD10 | AMACR | CK20 | Cathepsin-K | FH | SDHB | TFE3 |
| --- | --- | --- | --- | --- | --- | --- | --- | --- | --- | --- | --- | --- | --- |
|  |  |  |  |  |  |  |  |  |  |  |  |  |  |
| 1 | **1** | 3+ | 3+ | 0 | 3+ | 0 | 1+ | 0 | 0 | 1+ | 3+ | 3+ | 0 |
|  |  |  |  |  |  |  |  |  |  |  |  |  |  |
| 2 | **2** | 3+ | 3+ | 1+ | 3+ | 0 | 0 | 1+ | 0 | 0 | 3+ | 3+ | 0 |
|  |  |  |  |  |  |  |  |  |  |  |  |  |  |
| 3 | **3** | 3+ | 3+ | 0 | 3+ | 0 | 0 | 0 | 0 | 0 | 3+ | 3+ | 0 |
|  |  |  |  |  |  |  |  |  |  |  |  |  |  |
| 4 | **4** | 3+ | 3+ | 0 | 3+ | 0 | 1+ | 0 | 0 | 1+ | 3+ | 3+ | 0 |
|  |  |  |  |  |  |  |  |  |  |  |  |  |  |
| 5 | **5** | 3+ | 3+ | 1+ | 3+ | 0 | 0 | 0 | 0 | 0 | 3+ | 3+ | 0 |
|  |  |  |  |  |  |  |  |  |  |  |  |  |  |
| 6 | **6** | 3+ | 3+ | 0 | 3+ | 0 | 0 | 0 | 0 | 0 | 3+ | 3+ | 0 |
|  |  |  |  |  |  |  |  |  |  |  |  |  |  |
| 7 | **7** | 3+ | 3+ | 0 | 3+ | 0 | 0 | 1+ | 0 | 1+ | 3+ | 3+ | 0 |
|  |  |  |  |  |  |  |  |  |  |  |  |  |  |
| 8 | **8** | 3+ | 3+ | 1+ | 3+ | 0 | 0 | 0 | 0 | 1+ | 3+ | 3+ | 0 |
|  |  |  |  |  |  |  |  |  |  |  |  |  |  |
| 9 | **9** | 3+ | 3+ | 0 | 3+ | 0 | 0 | 0 | 0 | 0 | 3+ | 3+ | 0 |
|  |  |  |  |  |  |  |  |  |  |  |  |  |  |
| 10 | **10** | 3+ | 3+ | 1+ | 3+ | 0 | 0 | 0 | 0 | 0 | 3+ | 3+ | 0 |
|  |  |  |  |  |  |  |  |  |  |  |  |  |  |
|  | **11** | 3+ | 3+ | 0 | 3+ | 0 | 0 | 0 | 0 | 0 | 3+ | 3+ | 0 |
|  |  |  |  |  |  |  |  |  |  |  |  |  |  |
| 11 | **12** | 3+ | 3+ | 0 | 3+ | 0 | 0 | 1+ | 0 | 0 | 3+ | 3+ | 0 |
|  |  |  |  |  |  |  |  |  |  |  |  |  |  |
